# Importance of *Salmonella* Typhi-Responsive CD8+ T Cell Immunity in a Human Typhoid Fever Challenge Model

**DOI:** 10.3389/fimmu.2017.00208

**Published:** 2017-03-02

**Authors:** Stephanie Fresnay, Monica A. McArthur, Laurence S. Magder, Thomas C. Darton, Claire Jones, Claire S. Waddington, Christoph J. Blohmke, Brian Angus, Myron M. Levine, Andrew J. Pollard, Marcelo B. Sztein

**Affiliations:** ^1^Center for Vaccine Development, University of Maryland School of Medicine, Baltimore, MD, USA; ^2^Department of Epidemiology and Public Health, University of Maryland School of Medicine, Baltimore, MD, USA; ^3^Oxford Vaccine Group, Department of Paediatrics, University of Oxford, NIHR Oxford Biomedical Research Centre, Oxford, UK; ^4^Nuffield Department of Medicine, University of Oxford, Oxford, UK

**Keywords:** typhoid fever, *Salmonella* Typhi, cell-mediated immunity, CD8+ T cells, multifunctional, cytotoxicity, cytokines

## Abstract

Typhoid fever, caused by the human-restricted organism *Salmonella enterica* serovar Typhi (*S*. Typhi), constitutes a major global health problem. The development of improved attenuated vaccines is pressing, but delayed by the lack of appropriate preclinical models. Herein, we report that high levels of *S*. Typhi-responsive CD8+ T cells at baseline significantly correlate with an increased risk of disease in humans challenged with a high dose (~10^4^ CFU) wild-type *S*. Typhi. Typhoid fever development was associated with higher multifunctional *S*. Typhi-responsive CD8+ T effector memory cells at baseline. Early decreases of these cells in circulation following challenge were observed in both *S*. Typhi-responsive integrin α_4_β_7_− and integrin α_4_β_7_+ CD8+ T effector memory (T_EM_) cells, suggesting their potential to home to both mucosal and extra-intestinal sites. Participants with higher baseline levels of *S*. Typhi-responsive CD8+ T memory cells had a higher risk of acquiring disease, but among those who acquired disease, those with a higher baseline responses took longer to develop disease. In contrast, protection against disease was associated with low or absent *S*. Typhi-responsive T cells at baseline and no changes in circulation following challenge. These data highlight the importance of pre-existing *S*. Typhi-responsive immunity in predicting clinical outcome following infection with wild-type *S*. Typhi and provide novel insights into the complex mechanisms involved in protective immunity to natural infection in a stringent human model with a high challenge dose. They also contribute important information on the immunological responses to be assessed in the appraisal and selection of new generation typhoid vaccines.

## Introduction

Typhoid fever remains a major public health priority worldwide, with an estimated 21.7 million cases and 200,000 deaths per year (1). *Salmonella enterica* serovar Typhi (*S*. Typhi) is a human-restricted facultative intracellular Gram negative organism that causes typhoid fever (2). A better understanding of the immunological correlates of protection against *S*. Typhi is required for the development of improved attenuated typhoid vaccines. However, current knowledge is limited due to the difficulties associated with performing challenge studies in humans and the lack of an animal model that faithfully recapitulates human disease. Nevertheless, the *S*. Typhimurium “typhoid” mouse model has led to important insights into the role that various innate and adaptive effector mechanisms might play in protection from *Salmonella* infection, including production of interferon (IFN)-γ and tumor necrosis factor (TNF)-α by CD8+ T cells (3).

Human typhoid challenge studies were performed in the 1960s at the University of Maryland to improve understanding of typhoid fever (4, 5), and constituted a first step toward licensure of the oral attenuated Ty21a typhoid vaccine (6). However, due to the rudimentary immunological assays available at that time, this research did not document cellular-mediated immune responses (CMI) against *S*. Typhi and provided only very limited understanding of possible immunological correlates of protection. Significant information has been derived from studies that examined immune responses in typhoid patients after infection in the field and development of clinical typhoid disease or following vaccination with attenuated typhoid oral vaccines (7). However, these studies do not provide insights into the immunological status before wild-type infection and its possible effects on clinical outcome. The human challenge model was recently developed by the Oxford Vaccine Group (OVG, University of Oxford) where naïve participants ingested wild-type (wt) *S*. Typhi (Quailes strain) (8–10). The re-establishment of this challenge model allowed us, for the first time, to use advanced immunological tools to study the relationship between a subject’s pre-challenge immunologic status and subsequent clinical outcome following exposure to wt *S*. Typhi, as well as to initiate detailed studies of the immunological correlates of protection in typhoid fever (11).

A considerable body of literature in subjects immunized orally with Ty21a and attenuated typhoid vaccine candidates suggest that CMI responses, in particular CD8+ effector T cells, may play a crucial role in limiting the progression of typhoid fever by destroying host cells infected with *S*. Typhi (12, 13). CD8+ T cells may contribute to the control of infection through cytolytic activity and/or production of T helper 1/T cytotoxic 1, as well as Th17 cytokines (7, 11, 14–23). Multiphasic cytokine production by CD8+ T cells in participants immunized with live-attenuated typhoid vaccine Ty21a has been described in response to antigenic presentation by class Ia HLA and by non-classical HLA-E molecules, the latter molecule being less polymorphic and likely to present a more conserved set of bacterial peptides (7, 21, 22). Furthermore, detailed characterization of the simultaneous production of cytokines (co-production) by individual CD8+ T cells identified persistent *S*. Typhi-specific multifunctional (MF) CD8+ T cells following oral immunization with Ty21a (22). Finally, very recently, the closely monitored experimental human infection model with wt *S*. Typhi allowed us to provide the first evidence that CD8+ responses directed against *S*. Typhi correlate with clinical outcome in humans. This was observed in the group of participants challenged with a relatively low dose (~10^3^ CFU) of wild-type *S*. Typhi (11). Higher MF *S*. Typhi-responsive CD8+ T cells at baseline were associated with protection against typhoid and delayed disease onset. Moreover, following challenge, development of typhoid fever was accompanied by decreases in circulating *S*. Typhi-responsive CD8+ T effector memory (T_EM_) with gut homing potential, suggesting migration to the site(s) of infection. In contrast, protection against disease was associated with low or no changes in circulating *S*. Typhi-responsive T_EM_ (11).

In the present study, we characterized CMI responses directed against *S*. Typhi before pathogen exposure in participants from the Oxford study challenged with a high dose of wt *S*. Typhi (~10^4^ CFU). We evaluated the relationship between baseline levels of *S*. Typhi-responsive CD8+ T cells responses and clinical outcome. We also investigated the kinetic patterns of *S*. Typhi-responsive CD8+ T cells following challenge, as well as their expression of the gut homing molecule integrin α_4_β_7_ in relationship with typhoid diagnosis. Finally, we characterized in depth the MF properties of the responses against *S*. Typhi to identify the dominant MF patterns associated with clinical outcome by simultaneously evaluating the production of macrophage inflammatory protein (MIP)-1β, IFN-γ, TNF-α, interleukin (IL)-2, and IL-17A, as well as the expression of the cytotoxicity degranulation marker CD107a (24). The results described herein demonstrate that baseline responses directed against *S*. Typhi are related to clinical outcome following infection with a high dose (~10^4^ CFU) of wt *S*. Typhi in a stringent human challenge model.

## Materials and Methods

### Participants and Challenge

Twenty healthy, male or female participants aged 18–60 years were recruited by the Oxford Vaccine Group, UK, to participate in this phase II clinical study. The demographic characteristics of the participants are described in Table S1 in Supplementary Material. Any participant who had previously received typhoid vaccination, resided for over 6 months in typhoid-endemic areas, or was previously diagnosed with probable or confirmed typhoid infection was excluded from this study. The extensive exclusion criteria also included evidence of gallbladder disease, allergy to antibiotics, food handling, contact with susceptible individuals, acute or exacerbation of chronic infection within the previous 7 days or fever within the previous 3 days, and a history of having been treated with antibiotics or corticosteroids in the previous 14 days (8).

Participants were challenged orally with a dose of 1–5 × 10^4^ CFU of wt *S*. Typhi (Quailes strain) administered after neutralization of gastric acid with NaHCO_3_ and monitored closely as previously described (8). Positive typhoid fever diagnosis (TD) was determined by blood culture-confirmed *S*. Typhi bacteremia or development of a fever of ≥38°C for ≥12 h, and participants were treated as previously described (8). Written informed consent was obtained in accordance with the Declaration of Helsinki. All procedures were approved by National Research Ethic Service (NRES), Oxfordshire Research Ethics Comittee A (10/H0604/53) and conducted in accordance with the principles of the International Conference of Harmonisation Good Clinical Practice guidelines. All participants enrolled in this study [13 participants diagnosed with typhoid (TD) and 7 participants who were not diagnosed with typhoid (NoTD)] were evaluated for their T cell effector functions.

### Specimen Collection and Isolation of Peripheral Blood Mononuclear Cells (PBMC)

Routine blood hematology was performed before challenge, on alternate days after challenge, and at typhoid diagnosis. PBMC were isolated by Lymphoprep gradient centrifugation (STEMCELL Technologies, Vancouver, BC, Canada) and stored in liquid N_2_. Upon thawing, viability and recovery were measured as previously described (11), and cells were rested overnight in complete RPMI [cRPMI: RPMI 1640 media (Gibco, Carlsbad, CA, USA) supplemented with 100 U/ml penicillin (Sigma), 100 μg/ml streptomycin (Sigma), 50 μg/ml gentamicin (Gibco), 2 mM l-glutamine (Gibco), 10 mM HEPES buffer (Gibco), and 10% heat-inactivated fetal bovine serum (Gemini Bioproducts, West Sacramento, CA, USA)] to serve as effector cells in CMI assays.

### Stimulator Cells

Autologous Epstein–Barr virus (EBV)-transformed lymphoblastoid cell line (B-EBV cells) and autologous blasts were generated from the PBMC of each participant isolated before challenge. B-EBV cells were obtained by infection of PBMC with EBV particles [supernatant from the B95-8 cell line (ATCC CRL1612)] and cyclosporine (0.5 μg/ml; Sigma-Aldrich, Saint-Louis, MO, USA) for 15–30 days. Autologous blasts were prepared by 24 h incubating PBMC with 1 μg/ml PHA (Sigma-Aldrich, St. Louis, MO, USA) in cRPMI, followed by culture in cRPMI supplemented with 20 IU/ml recombinant human IL-2 (rhIL-2; Roche, Indianapolis, IN, USA) for 7 days. The B cell line 721.222.AEH, which is defective for HLA classical class I molecules but expresses non-classical class-I HLA-E molecules, was provided by Dr. D. Geraghty (20, 22) and cultured in cRPMI supplemented with 200 mU/ml hygromycin B (Sigma-Aldrich).

### *S*. Typhi Infection of Stimulator Cells

B-EBV cells, blasts, and AEH cells were incubated for 3 h at 37°C with wt *S*. Typhi strain ISP1820 (at a 7:1 bacteria:target ratio) in RPMI free of antibiotics. Cells were washed extensively with cRMPI and cultured overnight in cRPMI in the presence of gentamicin (150 μg/ml). Infection with *S*. Typhi was confirmed by flow cytometry after staining with anti-*Salmonella* common structural Ag (Kierkegaard & Perry, Gaithersburg, MD, USA) as previously described (12).

### *Ex Vivo* Stimulation of Effector Cells

Peripheral blood mononuclear cells were thawed and rested in cRPMI overnight before stimulation with *S*. Typhi-infected stimulating cells. Uninfected target (stimulator) cells and Staphylococcus enterotoxin B (SEB; 10 μg/ml) were used, respectively, as negative and positive controls. Stimulating cells were γ-irradiated (6,000 rad) and incubated with PBMC (effector: stimulator ratio 5:1) for 2 h in the presence of anti-CD107a (FITC, BD Biosciences) monoclonal antibody (mAb) before overnight incubation with the protein transport blockers monensin (1 μg/ml, Sigma) and brefeldin A (2 μg/ml; Sigma).

### Immunostaining with 14-Color Panel mAb and Flow Cytometry Analysis

After co-culture with stimulator cells, PBMC were harvested, washed in 1× PBS, stained extracellularly, permeabilized, and stained intracellularly, using a 14-color panel containing Yellow Live/Dead viability kit (Invitrogen, Carlsbad, CA, USA) and mAb to CD14-BV570 (M5E2, Biolegend), CD19-BV570 (HIB19, Biolegend), CD3-BV650 (OKT3, Biolegend), CD4-PECy5 (RPA-T4, BD), CD8-PerCP-Cy5.5 (SK1, BD), CD45RA-biotin (HI100, BD), CD62L-APC-A780 (DREG-56, Ebioscience), integrin α_4_β_7_-A647 (ACT1; conjugated in house) streptavidin(SAV)-Qdot800 (Invitrogen) CD69-ECD (TP1.55.3, Beckman Coulter), IFN-γ-PE-Cy7 (B27, BD), TNF-α-A700 (MAb11, BD), IL-2-BV605 (MQ1-17H12, Biolegend), IL-17A-BV421 (BL168, Biolegend), and MIP-1β-PE (IC271P, R&D) as previously described (11). Samples were acquired using a customized LSRII flow cytometer (BD Biosciences) and analyzed using Winlist v7.0 (Verity Software House, Topsham, ME, USA). Absolute numbers of CD3, CD8, CD4 positive cells and CD8 memory subsets cells were calculated by using percentages obtained from flow cytometry analysis related to the absolute number of lymphocytes determined by routine blood count. Responses against *S*. Typhi were expressed as net percentage of positive cells (i.e., total percentage of positive cells in the presence of *S*. Typhi-infected targets minus percentage of positive cells in co-cultures with uninfected cells).

### Statistical Analysis

Statistical analyses were performed as previously described for participants challenged with a low dose of wt *S*. Typhi (11). Mann–Whitney tests and linear regression analysis were performed using Prism v7.02 (GraphPad software, La Jolla, CA, USA). Areas under the curve were measured using the trapezoidal method (GraphPad Prism v7.02). *p* values <0.05 were considered significant. Some statistical analyses were based on multiple data points from the same individual as indicated in the text. For example, in some cases, the same individual provided information on cytokine production and/or CD107a expression levels after stimulation with three types of stimulations (EBV, AEH, and blasts), and these responses we evaluated with regard to clinical outcome (i.e., TD vs. NoTD patients). To take advantage of all this information in a single analysis, we used a mixed effects model fitted by maximum likelihood. The correlation between repeated measures on the same person was accounted for by including a random effect per person, using SAS 9.3 (Cary, NC, USA).

## Results

### Baseline—CD8+ T Cell Responses against *S*. Typhi Correlate with Clinical Outcome after Challenge

**Figure 1:**
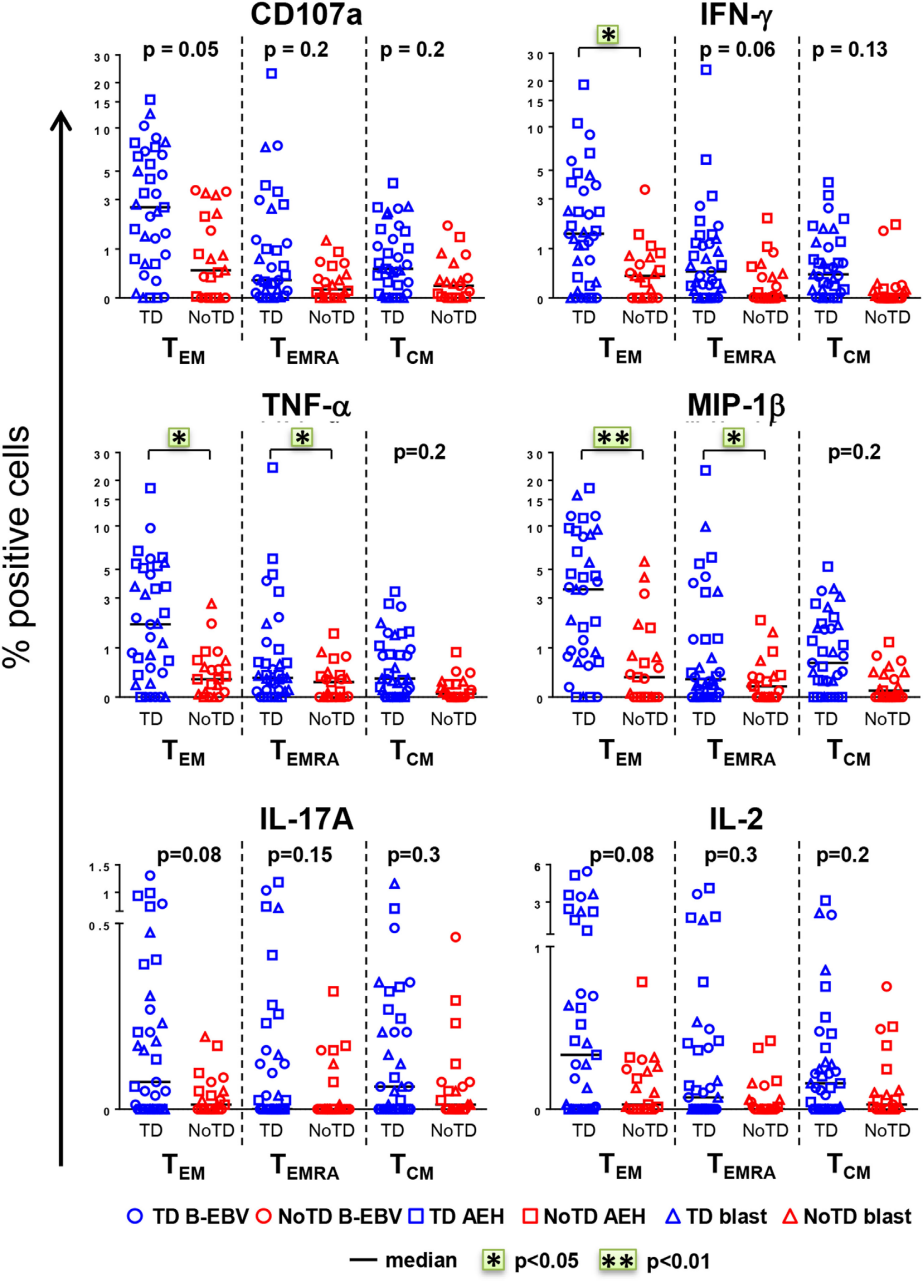
**CD8+ T cell responses against *Salmonella enterica* serovar Typhi (*S*. Typhi) at baseline predict the clinical outcome after challenge**. PBMC isolated at baseline from each participant (TD *n* = 13, blue; NoTD *n* = 7, red) were stimulated for 18 h with *S*. Typhi-infected AEH cells (squares), *S*. Typhi-infected B-EBV cells (circles), or *S*. Typhi-infected blasts (triangles). After co-culture, cells were immunostained with a 14-color panel of mAbs and analyzed by follow cytometry as described in Section “Materials and Methods.” Each symbol represents the net percentage of positive cells measured for CD107a, IFN-γ, TNF-α, MIP-1β, interleukin (IL)-17A, and IL-2 in the CD8+ T_EM_, T effector memory CD45RA+ (T_EMRA_), and T central memory (T_CM_) subsets as indicated. Statistical analyses were performed using mixed effects models to account for multiple observations per person. *p < 0.05; **p < 0.01.

The CD8+ T cell compartment is likely to play a major role in the CMI response against *S*. Typhi (7, 13–15, 18). Therefore, we first explored whether CD8+ T cell responses at baseline in healthy participants challenged with wt *S*. Typhi could predict subsequent typhoid diagnosis. PBMC were obtained from the 20 participants challenged with the high dose of wt *S*. Typhi (~10^4^ CFU), of whom 13 developed typhoid fever (TD group) and 7 did not (NoTD group). PBMC isolated before challenge (day 0) were stimulated *in vitro* with *S*. Typhi-infected autologous B-EBV, *S*. Typhi-infected autologous blasts, or *S*. Typhi-infected HLA-E-restricted AEH cells. Uninfected cells and SEB were used as negative and positive controls, respectively. PBMC were surface and intracellularly stained as described in Section “Materials and Methods.” Samples were analyzed by multichromatic flow cytometry, and CD8+ T cells were selected after a gating strategy involving the exclusion of dead cells and CD3-CD14+ CD19+ cells. Based on their expression of CD62L and CD45RA, CD8+ T cells were subsequently divided into naïve T (CD62L+ CD45RA+), T central memory (T_CM_; CD62L+ CD45RA−), T effector memory (T_EM_; CD62L−CD45RA−), and T_EM_ CD45RA+ (T_EMRA_; CD62L−CD45RA+). No differences were observed at baseline between the participants from the TD and NoTD groups in the absolute number of white cells, lymphocytes, CD3+, CD8+, CD4+, and CD8+ memory subsets (Figure S1A in Supplementary Material). Responses against *S*. Typhi were further characterized by co-expression of the T cell activation marker CD69 and the cytotoxicity degranulation marker CD107a, or the production of IFN-γ, TNF-α, MIP-1β, IL-17A, and/or IL-2. Most participants diagnosed with typhoid disease (TD) showed higher levels of *S*. Typhi-responsive T cells at baseline than participants who did not develop infection (NoTD) (Figure 1). Since these differences were observed after stimulation with each of three different *S*. Typhi-infected cells (Figure S1B in Supplementary Material), we therefore combined results from all stimulations for the analyses shown in Figure 1, as well as in some subsequent analyses as indicated below. Consistent with our previous findings (11), the T_EM_ subset represented the major source of intracellular chemokine/cytokine production and exhibited the highest expression of CD107a, followed by T_EMRA_ and T_CM_. Interestingly, in TD participants, CD8+ T_EM_ produced significantly higher levels of IFN-γ, TNF-α, and MIP-1β in response to *S*. Typhi stimulation than in NoTD participants. We also observed evidence of an association, albeit not reaching statistical significance, between development of typhoid disease and higher expression of CD107a and production of IL-17A and IL-2 in CD8+ T_EM_. Expression of CD107a and cytokine production in response to stimulation with *S*. Typhi-infected cells was also significantly higher (TNF-α, MIP-1β), or exhibited a strong trend (i.e., they did not reach statistical significance), in CD8+ T_EMRA_ and T_CM_ in the participants who developed disease. Interestingly, we did not observe statistically significant differences in baseline *S*. Typhi-responses between volunteers who developed typhoid disease defined by fever and positive bacterial cultures, and those who developed either fever or exhibited a positive bacterial culture.

**Figure 2:**
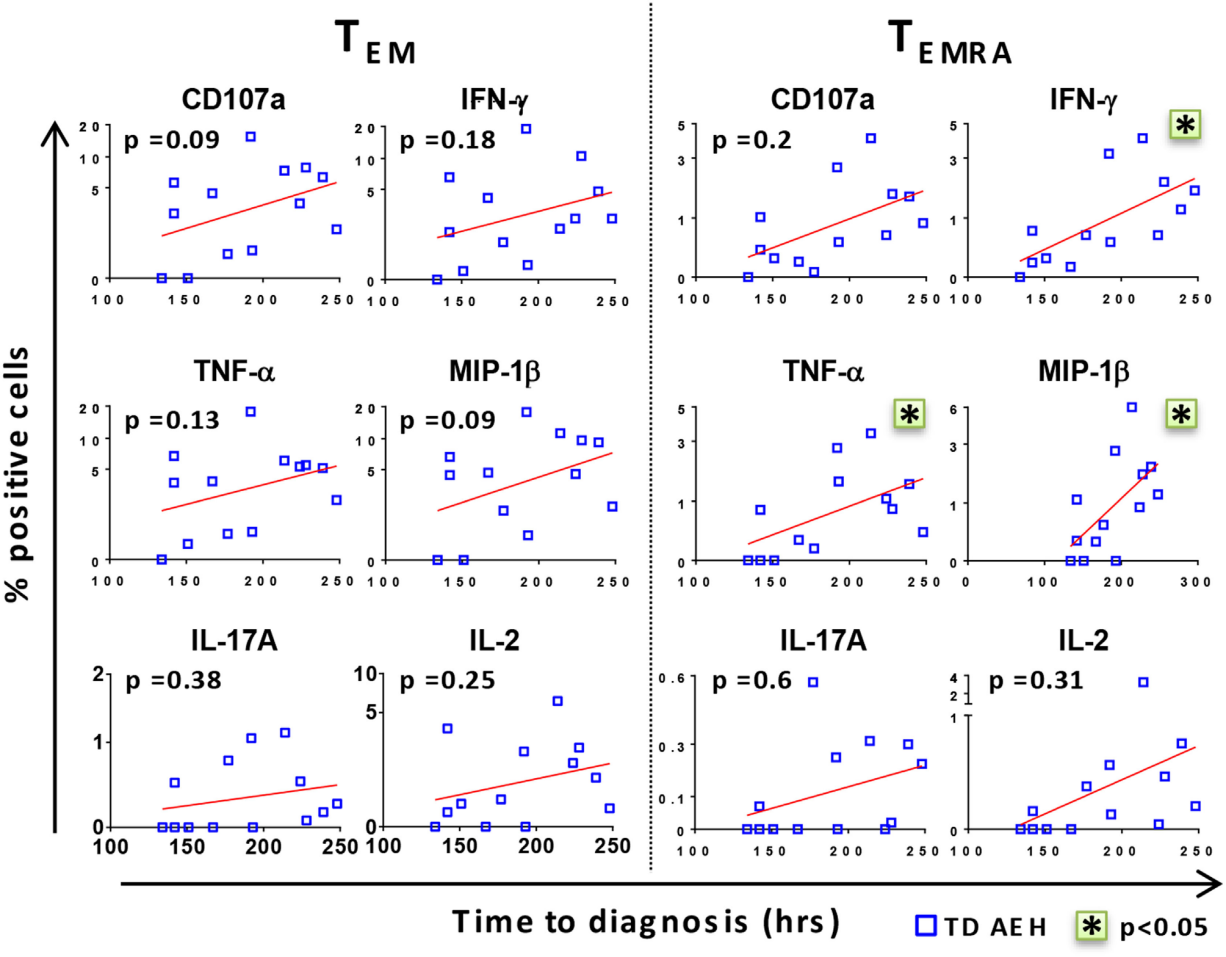
**High levels of CD8+ T cell responses against *Salmonella enterica* serovar Typhi (*S*. Typhi) are associated with delayed time to diagnosis**. After stimulation of PBMC with *S*. Typhi-infected AEH cells, percentages of CD107a, IFN-γ, TNF-α, MIP-1β, interleukin (IL)-17A, and IL-2-positive cells in the CD8+ T_EM_ and T effector memory CD45RA+ (T_EMRA_) subsets were plotted against time to diagnosis for each participant who developed typhoid fever (*n* = 13). Correlations (red lines) were assessed using the linear regression function and Pearson’s tests of GraphPad Prism v7.02. *p < 0.05.

After identifying that the level of baseline responses directed against *S*. Typhi was associated with clinical outcome upon challenge, we investigated whether there was a relationship between these baseline levels and the time to disease diagnosis in TD participants. Interestingly, we found a positive trend between high baseline levels of all markers and delay in time of disease onset (Figure 2). Stronger correlations were observed in the T_EMRA_ subset after stimulation with *S*. Typhi-infected AEH cells, where the production of IFN-γ, TNF-α, and MIP-1β before challenge and the time to diagnosis were significantly correlated (Figure 2). These data indicate that the presence of higher levels of *S*. Typhi-responsive T cells before challenge among the participants who develop typhoid fever was associated with a delayed onset of typhoid fever, suggesting that these T cells could delay, but ultimately could not prevent, development of disease.

### Distinct Clinical Outcomes Are Accompanied by Discrete Responses against *S*. Typhi after Challenge

**Figure 3:**
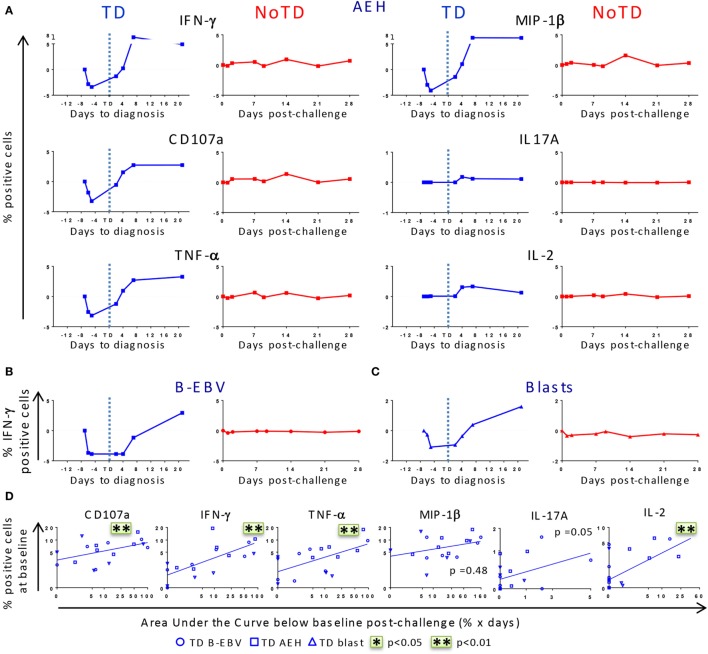
**Distinct kinetics of responsive CD8+ T cell responses against *Salmonella enterica* serovar Typhi (*S*. Typhi) after challenge**. **(A)** Kinetics for a representative participant from each group (TD and NoTD) showing the production of cytokines/chemokines and expression of CD107a by CD8+ T_EM_ following stimulation with *S*. Typhi-infected AEH cells at different time points after challenge. **(B,C)** Kinetics of IFN-γ production is shown for a representative volunteer after stimulation with *S*. Typhi-infected B-EBV cells and *S*. Typhi-infected blasts. **(D)** Areas under the curve below baseline were measured until time to diagnosis for the six randomly selected TD participants and plotted against the baseline level of responses for each of the indicated biomarkers stimulated with *S*. Typhi-infected AEH cells (squares), *S*. Typhi-infected B-EBV cells (circles), or *S*. Typhi-infected blasts (triangles). Correlations analyses were performed using mixed effects models to account for multiple observations per person. **p < 0.01.

After identifying that differences in baseline responses were correlated with clinical outcome, we studied the kinetics of immune responses until day 28 after challenge. PBMC were obtained from 12 randomly selected participants, 6 of whom developed typhoid fever (TD group) and 6 who did not (NoTD group). Consistent with the decreased lymphocyte counts previously reported for the TD participants (8), we observed a drop of the absolute numbers of CD3+, CD8+, CD4+ T cells before diagnosis (Figure S2A in Supplementary Material). We next investigated whether challenge with wt *S*. Typhi elicited multiphasic responses such as those observed after vaccination with Ty21a (21, 22). Because of the variable responsiveness at baseline in the different participants, the net values (day × post-challenge − day 0) were used to normalize the data. After challenge, five out of six TD participants showed a pronounced decrease in expression of CD107a and production of IFN-γ, TNF-α, and MIP-1β in all T_M_ subsets following stimulation with *S*. Typhi-infected AEH cells, while only minor changes were observed in IL-2 and IL-17A production (Figure 3A; Figure S3A in Supplementary Material). While only a small decrease was observed in the absolute numbers of CD8+ T_EM_, the decrease was striking in absolute numbers of *S*. Typhi-responsive T_EM_ expressing CD107a or producing IFN-γ (Figures S2A,B in Supplementary Material). This early drop was followed by a sharp rebound above baseline levels after disease onset. In contrast, responses for all biomarkers were negligible in five out of the six NoTD participants (Figure S3A in Supplementary Material). These distinct kinetic patterns were observed consistently after stimulation with all the *S*. Typhi-infected cell types (AEH cells, B-EBV cells, or blasts) (Figures 3A–C). However, despite the general similarity of the kinetic patterns observed in the TD group, variability in the magnitude of the decrease after challenge was noticed among participants (Figure S3A in Supplementary Material). To account for this variability, we measured the area under the curve below baseline for all early time points, and investigated whether the magnitude of the decrease in response to *S*. Typhi-infected cells was related to the baseline levels. A positive trend was noted for all functions in each of the T_M_ subsets, and significant correlations were observed for CD107a, IFN-γ, TNF-α, and IL-2 in T_EM_ cells (Figure 3D). Of note, T_EM_ cells showed a more pronounced decrease for most functions, followed by T_EMRA_ and T_CM_ cells (Figure S3B in Supplementary Material).

### Enhanced Gut Homing Potential of *S*. Typhi-Responsive CD8+ T Cells in Participants Diagnosed with Typhoid Disease

**Figure 4:**
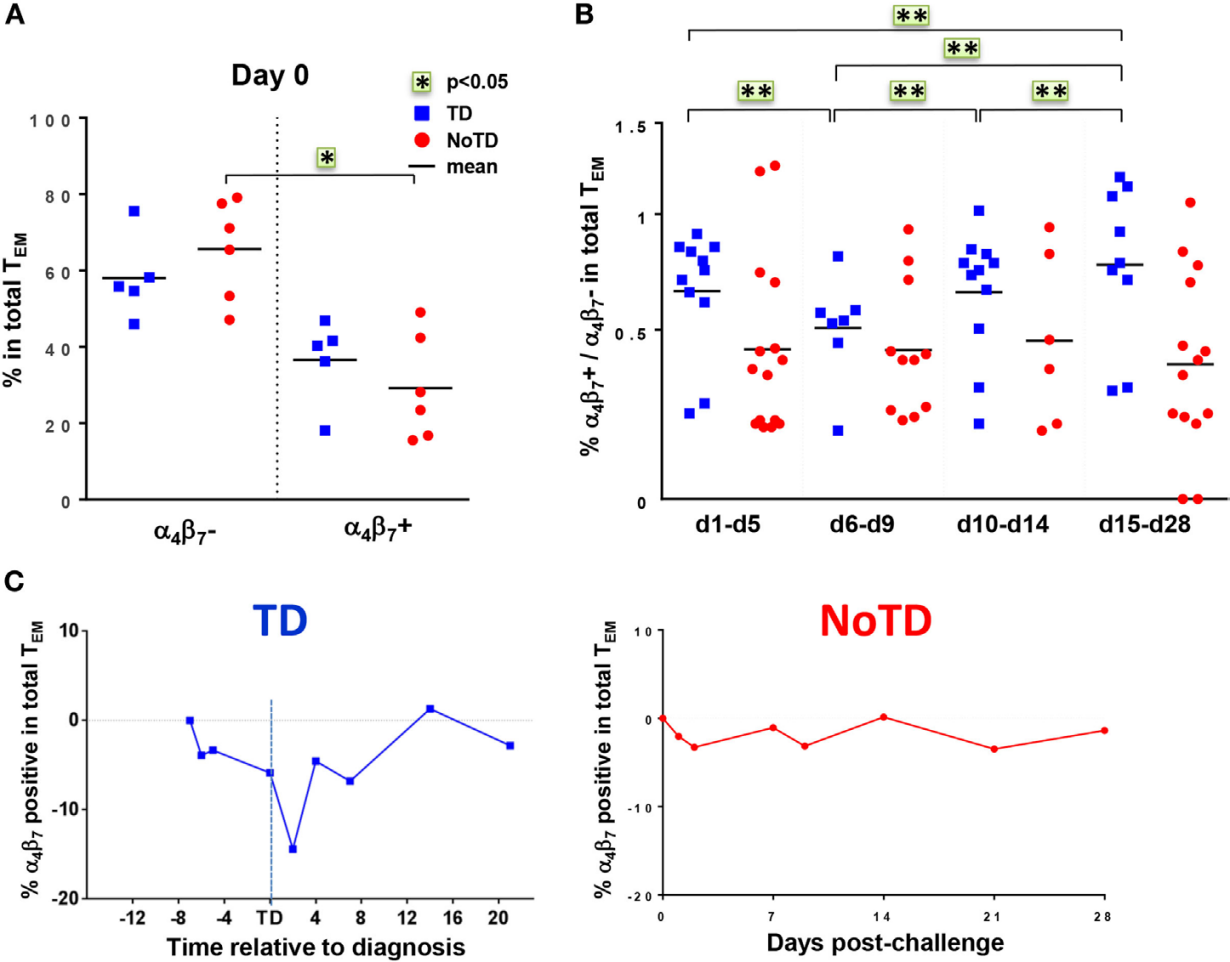
**Gut homing potential of total CD8+ T_EM_**. **(A)** Each value represents the baseline percentages of integrin α_4_β_7_− and integrin α_4_β_7+_ cells in total CD8+ T_EM_ evaluated by surface staining and flow cytometry for 12 randomly selected TD (squares) and NoTD (circles) participants. *p < 0.05. **(B)** Ratio of integrin α_4_β_7_+ to integrin α_4_β_7_− total CD8+ T_EM_ cells are shown within four periods of time after challenge. Period d6–d9 corresponds to the time of typhoid diagnosis. Statistical analyses were performed using mixed effects models to account for multiple observations per person. **p < 0.01. **(C)** Kinetics of expression of integrin α_4_β_7_+ total CD8+ T_EM_ after challenge in representative participants from each group.

The mucosal immunity mounted in the gut microenvironment, the site of entry for *S*. Typhi, is a major element in the protection against typhoid fever after vaccination with live oral typhoid vaccines (16, 19). Therefore, we sought to assess if the selective migration of CD8+ T cells to the small intestine, driven by the gut homing molecule integrin α_4_β_7_, was potentially contributing to the decrease in responses against *S*. Typhi observed soon after challenge. PBMC were obtained at baseline from the 20 participants (13 TD and 7 NoTD participants), and after challenge from the 12 randomly selected participants (6 from TD group and 6 from NoTD group). We first measured the expression of integrin α_4_β_7_ *ex vivo*, in total CD8+ T_EM_ cells, and found no difference in the baseline proportion of integrin α_4_β_7_ expressing cells between the two groups of participants (Figure 4A). In both groups, the percentages of integrin α_4_β_7_+ CD8+ T_EM_ cells represented approximately one third of the total CD8+ T_EM_ cells. Interestingly, a decline in the proportion of integrin α_4_β_7_+ CD8+ T_EM_ was observed around the time of diagnosis (d6–d9) for TD participants, while this proportion remained unchanged for NoTD participants (Figures 4B,C).

**Figure 5:**
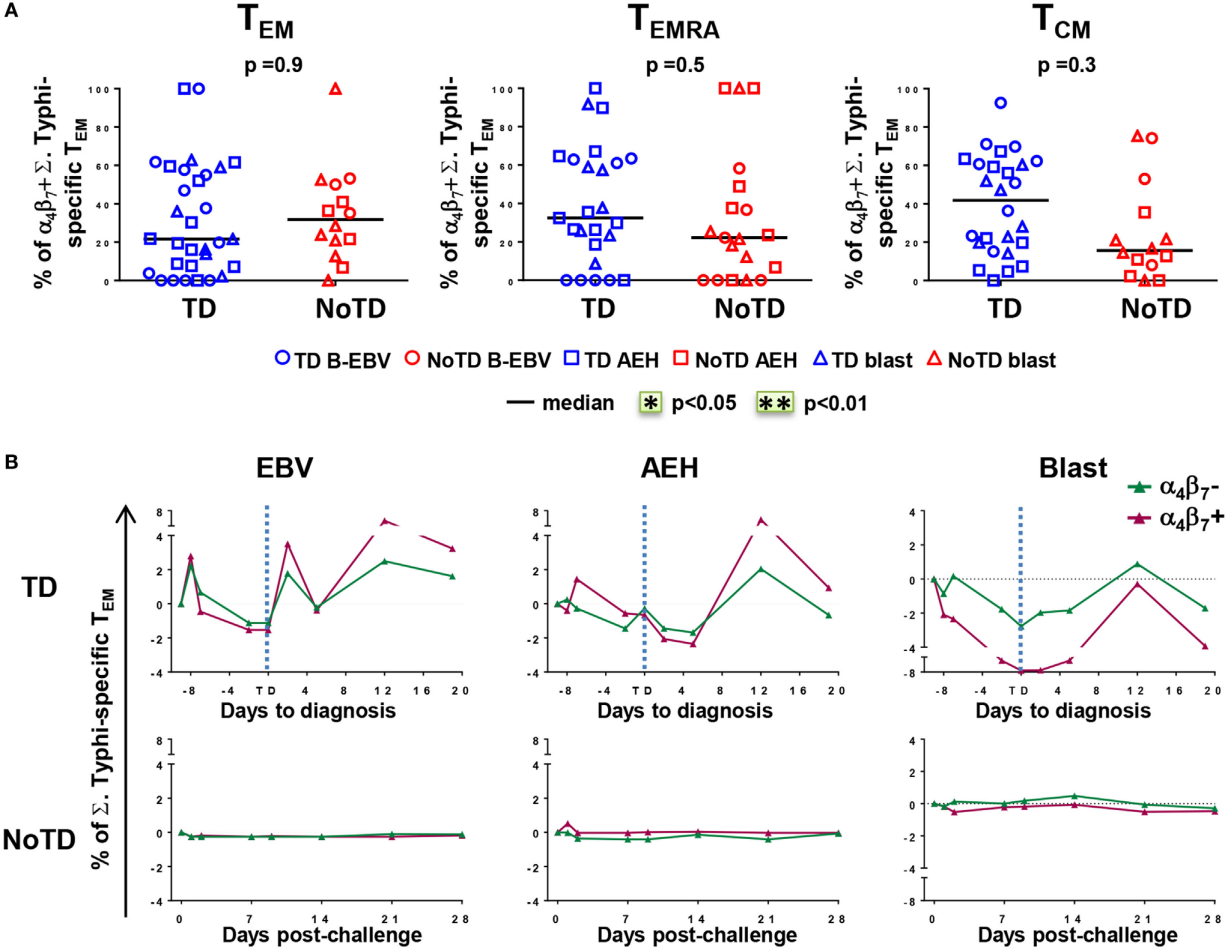
**Gut homing potential of *Salmonella enterica* serovar Typhi (*S*. Typhi)-responsive memory CD8+ T cell subsets**. **(A)** Each value represents the percentage of integrin α_4_β_7_+ cells in *S*. Typhi-responsive CD8+ T_EM_, T effector memory CD45RA+ (T_EMRA_), and T central memory (T_CM_) cell subsets at baseline for each participant (TD *n* = 13, NoTD *n* = 7) following stimulation with *S*. Typhi-infected cells [AEH cells (squares), B-EBV (circles), or blasts (triangles)]. **(B)** Kinetics of the percentages of integrin α_4_β_7_− and integrin α_4_β_7_+ *S*. Typhi-responsive CD8+ T_EM_ are shown for a representative participant in each group. Statistical analyses were performed using mixed effects models to account for multiple observations per person.

We next measured the expression of integrin α_4_β_7_ on *S*. Typhi-responsive activated (CD69+) T_M_ subsets after stimulation with *S*. Typhi-infected cells (AEH cells, B-EBV cells, or blasts). We observed no differences in the percentages of integrin α_4_β_7_− and integrin α_4_β_7_+ activated T_EM_ cells between the two groups of participants at baseline (Figure 5A). Early after challenge, TD participants showed similar decreases in both integrin α_4_β_7_− and integrin α_4_β_7_+ *S*. Typhi-responsive T_EM_, and both populations rebounded over baseline after disease diagnosis (Figure 5B). No changes were observed in NoTD participants.

### *S*. Typhi-Responsive CD8+ T_M_ Cells Are Primarily MF

**Figure 6:**
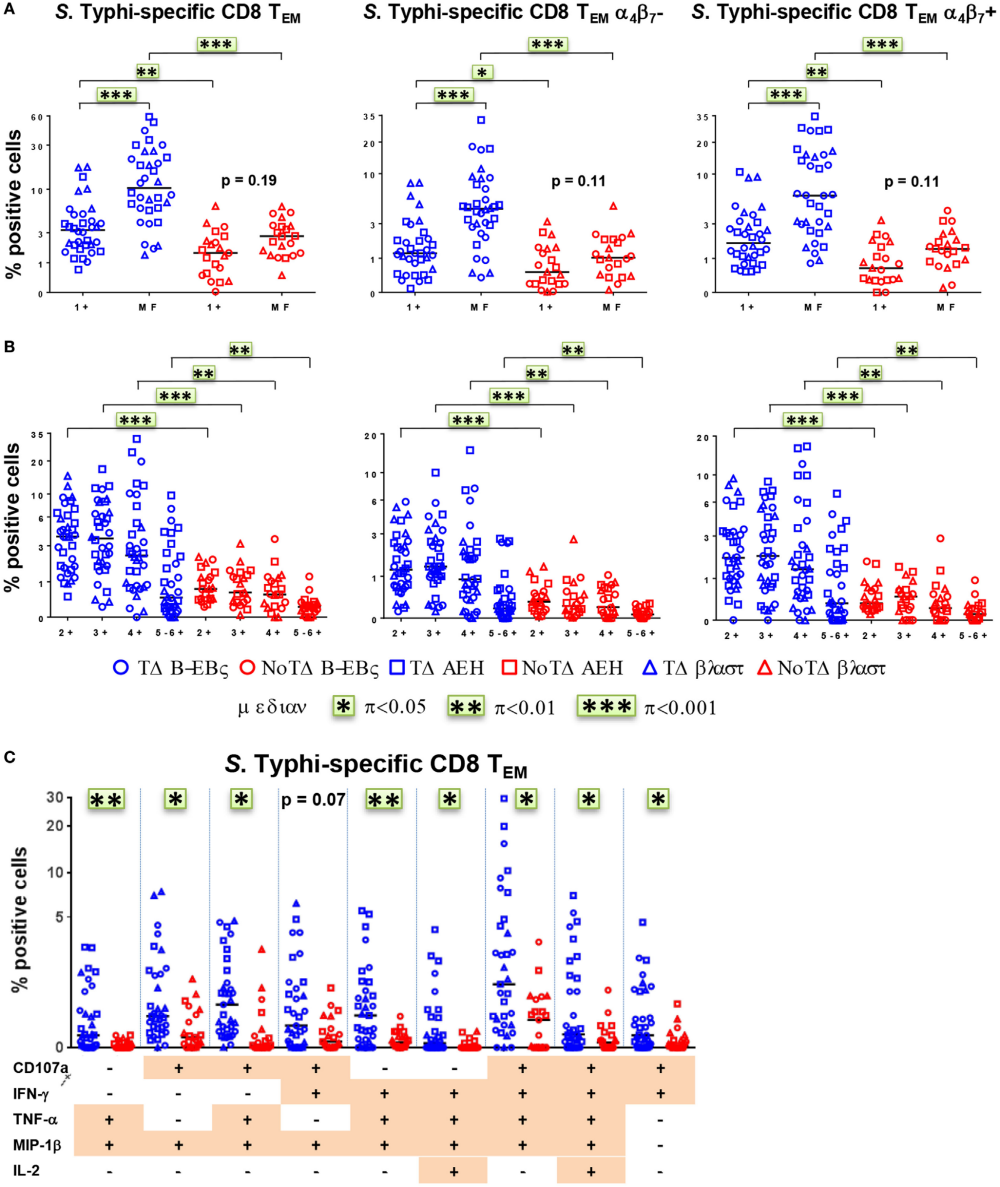
**Characterization of multifunctional (MF) CD8+ T_EM_ responses against *Salmonella enterica* serovar Typhi (*S*. Typhi) at baseline**. Flow cytometry data were analyzed using the FCOM function of Winlist to determine the proportion of all possible combinations of the six measured biomarkers to identify MF cells (i.e., positive for several biomarkers concomitantly). Each symbol represents the percentage of the different populations measured after stimulation with *S*. Typhi-infected cells [AEH cells (squares), B-EBV (circles) cells, or blasts (triangles)] for each participant (TD *n* = 13, NoTD *n* = 7). **(A)** The percentages of single positive cells (1+) or of total MF cells (i.e., the sum of all cells concomitantly positive for two or more biomarkers) are represented for all CD8+ T_EM_ and for CD8+ T_EM_ integrin α_4_β_7_− and integrin α_4_β_7_+ cells. **(B)** MF cells were divided into four groups on the basis of the number of biomarkers they expressed [e.g., cells expressing two biomarkers are shown as double positive (2+)]. **(C)** Characterization of the nine major individual populations of *S*. Typhi-responsive MF in CD8+ T_EM_. *p < 0.05, **p < 0.01, ***p < 0.001.

We and others have recently described that concomitant production of cytokines/chemokines and/or expression of CD107a at the single cell level (MF cells) are likely to be a critical factor in shaping the quality of protective immune responses (11, 21, 22, 25, 26). Therefore, we closely examined the proportion and characteristics of all possible combinations of the six functional biomarkers (i.e., a total of 64 possible combinations) measured in CD8+ T_EM_ cells. PBMC were obtained at baseline from the 20 participants (13 TD and 7 NoTD), and after challenge from 12 randomly selected participants (6 TD and 6 NoTD). For ease analysis, we first grouped them into single positive (1+) or MF cells, i.e., cells positive for two or more biomarkers (Figure 6A). At baseline, the MF populations were significantly higher in participants diagnosed with typhoid disease (Figure 6A). This was also observed in the numbers of absolute numbers of single and MF cells (Figure S4A in Supplementary Material). In addition, to explore the gut homing potential of these *S*. Typhi-responsive MF T cells, we measured their expression of integrin α_4_β_7_. We observed that the proportions of MF were similar in both integrin α_4_β_7_− and integrin α_4_β_7_+ T_EM_ (Figure 6A). To further characterize the MF responses, we categorized them into double (2+), triple (3+), quadruple (4+), and quintuple-sextuple (5–6+) positive subsets, based on the number of biomarkers they exhibited. We observed that double, triple, and quadruple positive populations comprise the dominant populations of MF CD8+ T_EM_ in TD and NoTD participants (Figure 6B). However, these populations were significantly higher in the participants who developed disease. Of note, these MF cells were equally represented by integrin α_4_β_7_− and integrin α_4_β_7_+ expressing T_EM_ subsets. We further focused on the precise characteristics of the individual MF populations and identified the nine dominant (i.e., highest frequency) MF patterns among all possible combinations (Figure 6C). Together, these nine populations represented 65–85% of the total *S*. Typhi-responsive MF cells. Interestingly, eight out of the nine distinct populations were significantly higher in participants who were diagnosed with typhoid disease. The production of the chemokine MIP-1β was a common feature as it was produced by eight out of the nine MF populations. CD107a, IFN-γ, and TNF-α were also present in six out of nine populations, while IL-2 was detected at much lower frequencies. The CD107a+IFN-γ+TNF-α+MIP-1β+ population was predominant, followed by four subdominant populations defined as CD107a+TNF-α+MIP-1β+, CD107a+IFN-γ+MIP-1β+, IFN-γ+TNF-α+MIP-1β+, and CD107a+IFN-γ+TNF-α+MIP-1β+IL-2+. IL-17A was detected at lower levels and was not produced by any of the nine dominant populations. The nine dominant populations were also equally represented in integrin α4β7− and integrin α4β7+ expressing T_EM_ subsets (Figure S4B in Supplementary Material).

**Figure 7:**
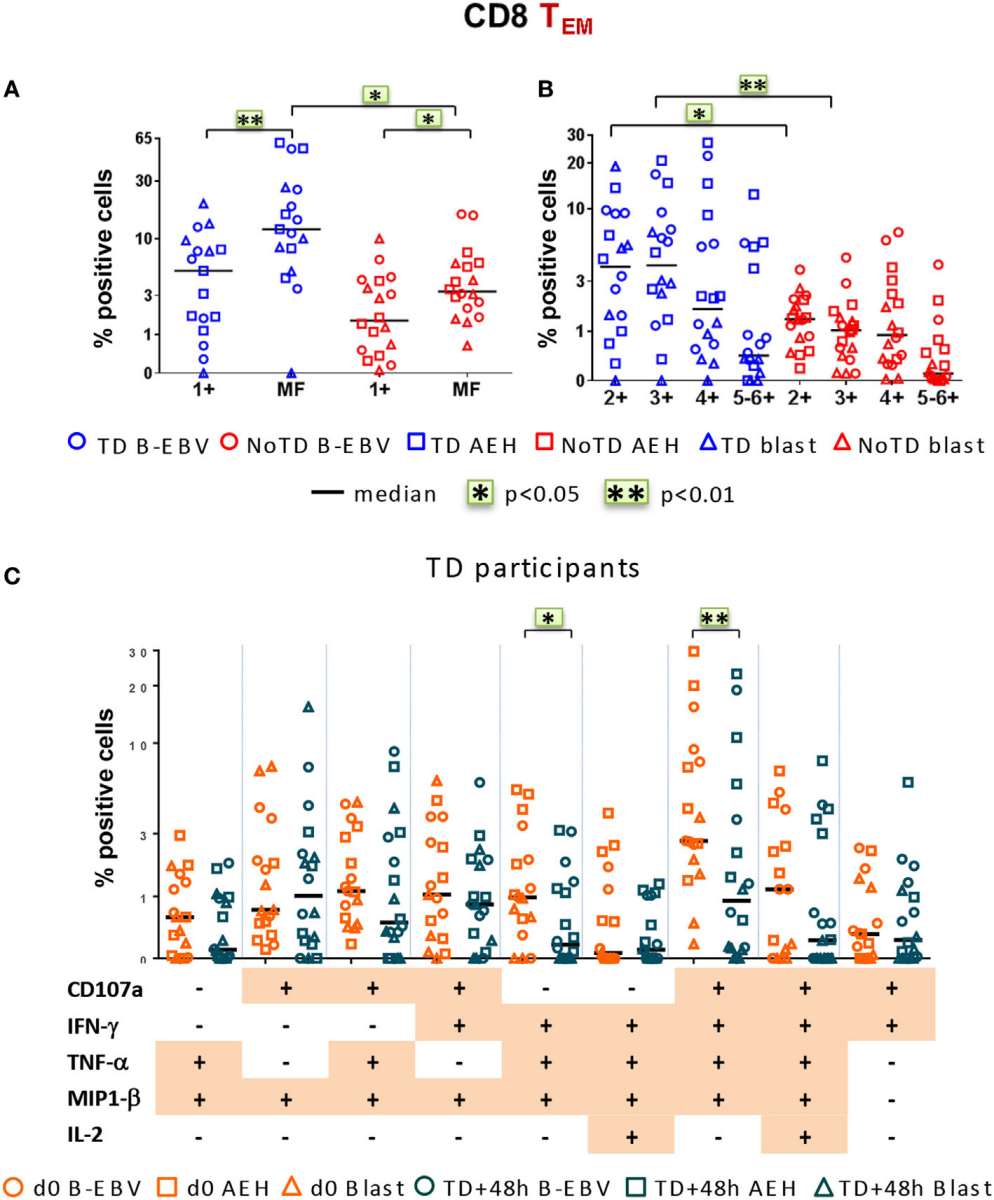
**Characterization of multifunctional (MF) CD8+ T_EM_ responses against *Salmonella enterica* serovar Typhi (*S*. Typhi) after challenge**. Flow cytometry data were analyzed as described in Figure 6. Percentages were measured at day 7 for six NoTD participants and at 48 h after typhoid diagnosis for six TD participants. **(A)** Percentages of single positive cells (1+) or of total MF cells. **(B)** Total MF cells were divided into four groups on the basis of the number of biomarkers they expressed. **(C)** The nine major individual populations of MF in TD participants are represented at baseline (orange) or 48 h after TD diagnosis (dark green).

Finally, we investigated post-challenge responses directed against *S*. Typhi at 48 h after typhoid diagnosis or day 7 post challenge in TD and NoTD participants, respectively, and found that, similar to baseline, MF responses were dominant (Figure 7A). Double, triple, and quadruple positive populations were the dominant MF cells (Figure 7B). Significant decreases in the percentages of only two of the nine dominant MF populations, i.e., CD107a+IFN-γ+TNF-α+MIP-1β+ and IFN-γ+TNF-α+MIP-1β+, occurred 48 h after typhoid diagnosis compared to baseline (Figure 7C). The dominance of MF populations was similar in both integrin α_4_β_7_− and integrin α_4_β_7_+ *S*. Typhi-responsive T_EM_ subsets (Figures S5A–C in Supplementary Material). Taken together, these results highlight a strong MF component of CD8+ T_EM_ responses, both at baseline and after challenge.

## Discussion

The investigation of immune responses in typhoid fever, particularly CMI, has been largely restricted to studies in endemic areas or following vaccination. Very limited data are available on the *S*. Typhi-specific immune status prior to infection with wt *S*. Typhi or on the immune correlate(s) of protection. This challenge study allowed us to investigate relationships between baseline levels of responses directed against *S*. Typhi and the development of typhoid disease. We have very recently published a description of the CMI responses for the cohort of participants challenged with a low dose (~10^3^ CFU) of wt *S*. Typhi (11). Herein, we report the results of CMI studies in participants challenged with a high dose of wt *S*. Typhi (~10^4^ CFU) that show higher baseline responses against *S*. Typhi to be associated with typhoid fever diagnosis. After challenge, TD participants showed decreases in both the percentages of *S*. Typhi-responsive CD8+ cells and absolute numbers of CD3+, CD8+, and CD4+ T cells, an observation consistent with the reported drop of lymphocyte counts described before diagnosis in TD participants (8) and with our previous findings in the group of participants challenged with the lower dose of *S*. Typhi (11).

Selective homing to the gut, the site of entry for *S*. Typhi, is driven to a large extent by the expression of the gut homing molecule integrin α_4_β_7_ (18, 27–30). In the present study, TD participants show decreases in both *S*. Typhi-responsive integrin α_4_β_7_− and integrin α_4_β_7_+ CD8+ T_EM_ cells after challenge, suggesting that migration of the CD8+ T_EM_ might be associated with the development of typhoid fever and that *S*. Typhi-responsive T_EM_ cells migrate not only to mucosal sites but also to other sites, presumably other RES components (e.g., peripheral lymph nodes, spleen). Their presence in secondary lymphoid tissues may restrict replication of *S*. Typhi during the incubation phase, delaying the onset of disease. These results are similar to those that we recently reported in the group of Oxford participants challenged with the lower dose of *S*. Typhi (11) and are in agreement with our previous observations after vaccination (16, 18, 19).

Interestingly, studies of acute *S*. Typhimurium infection in mouse models have shown that pathogenic bacteria can exploit inflammation in order to overcome the protective microbiota (31, 32). In contrast to the findings, we reported in the cohort of TD participants challenged with the lower dose of *S*. Typhi (11), among TD participants who received a higher inoculum those with higher baseline responses against *S*. Typhi were more likely to acquire disease. The contrasting association of clinical outcome and levels of *S*. Typhi-responsive CD8+ responses observed between the two groups of participants (i.e., those challenged with 10^3^ vs. 10^4^ CFU of wt *S*. Typhi) could be related to the presence of a larger number of wt *S*. Typhi organisms in the gastrointestinal mucosa in the group receiving 10^4^ CFU of wt *S*. Typhi. Although the data currently available do not allow us to fully explain these observations, it is reasonable to speculate that this larger numbers of *S*. Typhi organisms triggered a stronger inflammatory response than that induced with the lower inoculum and that this increased inflammation could favor the systemic spread of *S*. Typhi, ultimately leading to the development of typhoid fever. Moreover, the migration of large numbers of *S*. Typhi-responsive CD8+ T cells to the sites of infection might also play a role in the inflammatory responses. Additional information will be provided by ongoing studies directed to extend the current observations, which are based on a relatively small number of volunteers. These studies involve volunteers who participated in a follow-up clinical trial in which they were immunized, or not, with *S*. Typhi vaccines and then challenged with a high dose of wt *S*. Typhi.

Of great importance, we also show that higher classical and non-classical (HLA-E) class I MHC-restricted *S*. Typhi-specific baseline responses against *S*. Typhi among TD participants were associated with a delayed time to diagnosis, suggesting that *S*. Typhi-responsive CD8+ responses play a role in protection. These decreases in circulating *S*. Typhi-responsive T cells after challenge are proportional to the levels present at baseline, suggesting that the higher the pool of *S*. Typhi-responsive T cells available in circulation at baseline, the higher the number of these cells that are recruited to the site of pathogen encounter. Similar observations were reported in the group of participants challenged with the lower dose of wt *S*. Typhi (11). Based on these observations, we hypothesize that in TD participants the numbers of *S*. Typhi-responsive T cells recruited to the gut are sufficient to reach the threshold of inflammation necessary for *S*. Typhi to spread, but only participants with the highest numbers of *S*. Typhi-responsive T cells were able to initially delay *S*. Typhi dissemination, likely by decreasing the number of infectious organisms. Alternatively, or in addition, it is likely that the balance between suppressive and pro-inflammatory responses might also be important in the host’s ability to mount effective immune responses.

Exposure to wt *S*. Typhi might elicit *S*. Typhi-responsive regulatory T cells (T_reg_), which could suppress *S*. Typhi-responsive T_EM_ responses and contribute to the development of typhoid disease. This hypothesis is supported by studies in the murine *S*. Typhimurium model showing that the balance of suppressive T_reg_ and pro-inflammatory T cell responses influence bacterial clearance or persistence (33). We have recently described an upregulation of the gut homing molecule integrin α4β7 on *S*. Typhi-responsive T_reg_ in TD participants pre-challenge, followed by a significant downregulation post-challenge (34), suggesting possible T_reg_ homing to the gut. Additionally, *S*. Typhi-responsive T_reg_ from TD participants exhibited an upregulation of activation molecules post-challenge and *in vitro* depletion of T_reg_ resulted in increased cytokine production by CD8+ T_EM_. These results suggest that activated T_reg_ may play a pivotal role in typhoid fever, possibly through suppression of *S*. Typhi-responsive effector T cell responses (34). It is unclear whether similar T_reg_ responses were also elicited in the participants challenged with the low dose of wt *S*. Typhi and whether they could account, at least in part, for the differences observed in T effector baseline responses and clinical outcome between participants who received the low and high doses of wt *S*. Typhi.

It is also possible that other functional characteristics of *S*. Typhi-specific cells at baseline are important in defining the clinical outcome following challenge. Among these, the levels of exhausted *S*. Typhi-specific cells at baseline could be an important determinant. Exhausted T cells express the programmed death-1 receptor and are characterized by poor effector function that prevents optimal control of infections (35, 36). In our MF analysis, we noted that IFN-γ and TNF-α [typically expressed by PD1+ cells (37)] and which are potent inducers of PD-Ligand1 (38) were co-expressed in the dominant MF populations of *S*. Typhi-responsive T cells. Ongoing studies directed to assess the levels and functional properties of PD-1+ *S*. Typhi-specific cells in participants vaccinated with Ty21a, or not, and subsequently challenged with wt *S*. Typhi will help establish the validity of this hypothesis. In addressing the immunological differences observed between low and high doses of wt *S*. Typhi and their association with clinical outcome, we should also consider that in this stringent high-dose controlled human infection study, participants were exposed to high doses of pathogen, which are likely to exceed the number of infectious *S*. Typhi organisms expected to be ingested in the natural environment and which could have overwhelmed the host’s ability to successfully combat infection.

Numerous studies suggest that *S*. Typhi has the ability to reduce intestinal inflammation by several mechanisms including regulation of the innate immune signaling pathways (39), escape from recognition by innate immune cells (40), inhibition of antigen presentation by dendritic cells (41), or direct inhibition of T cells (42, 43). The possibility that *S*. Typhi might escape immune recognition and that this might play a key role in limiting inflammatory responses is supported by the fact that a few participants in this study were bacteremic without any reported symptoms. Similar observations were previously described in the Maryland challenges (5).

In this study, we provide additional information that the CD8+ T_EM_ subset is the major effector in responses against *S*. Typhi and show that these responses are mostly MF. These observations extend previous studies in humans and non-human primates demonstrating that the quality of the MF response is likely to play a critical role in immune responses against *S*. Typhi (25, 26, 44–46). Production of MIP-1β is a leading feature of *S*. Typhi-responsive MF populations, including the most dominant MF subset, i.e., CD107a+IFN-γ+TNF-α+MIP-1β+ cells. MIP-1β exerts a crucial role in tilting immune responses toward inflammation (47). In addition, MIP-1β has also been shown to be involved in CTL activity and its expression by HIV antigen-responsive CD8+ T cells in non-progressors suggests that it might play a role in controlling infection (46). In *S*. Typhi infections we previously described the co-production of MIP-1β with IFN-γ, TNF-α, IL-17A, and IL-2, as well as expression of CD107a following vaccination with Ty21a (22, 48). MIP-1β was also reported to be produced by PBMC obtained from *S*. Typhi-infected convalescent patients (49). Taken together, these observations suggest that coproduction of MIP-1β with other cytokines is a key component of responses against *S*. Typhi-and that, depending on the characteristics of the response (e.g., production of other cytokines/chemokines, kinetics, magnitude, microenvironment, other unknown factors), it may either play a key role in protection or in the development of inflammation leading to typhoid disease.

We observed that protection against typhoid fever is associated with very low or no baseline responses against *S*. Typhi and no changes in circulating *S*. Typhi-responsive T_M_ after challenge. These kinetic patterns were also consistently observed in the NoTD participants challenged with a lower dose (11). It is reasonable to hypothesize that in NoTD participants *S*. Typhi was controlled by innate and/or adaptive immune responses in the mucosa of the gastrointestinal tract, precluding *S*. Typhi from becoming invasive and causing disease. Although the mechanisms underlying the control of *S*. Typhi infection remain unclear, mucosal-associated invariant T (MAIT) cells may be one of the cell types involved. MAIT cells are a CD8+ cell subset expressing CD161 and TCR Vα7.2 and restricted by the MR1 MHC-related molecule, which has been postulated to play an important role in mucosal immunity (50). Our recent observations show that MAIT cells isolated from healthy individuals not previously exposed to *S*. Typhi are able to produce IL-17A, IFN-γ, and TNF-α when exposed to *S*. Typhi-infected cells (51). Conventional antigen-responsive T cells such as tissue-resident memory T cells, TCRγ/δ T cells, and NK-T cells may also help to provide protection in the gut microenvironment (52). Measurements of MAIT cell responses, as well as those of other subsets, in the challenged participants before and after exposure to wt *S*. Typhi will help clarify their role in typhoid fever.

In contrast to our observations on CD8+ baseline responses, higher baseline titers of antibodies directed against *S*. Typhi were not associated with development of disease (8). However, increases in *S*. Typhi-specific LPS and H antibodies post-challenge were associated with typhoid fever, while little change was seen in participants who did not develop disease. Further studies will be required to fully characterize the role of anti-*S*. Typhi antibodies in typhoid fever.

Since very few data are available on the baseline immune responses in the context of typhoid fever, the reasons for the disparity observed between baseline responses against *S*. Typhi and clinical outcome between the low and high dose participants are unclear. Participants were recruited in the UK, a non-endemic region (i.e., it is very unlikely that they have previously encountered *S*. Typhi), and they have not been vaccinated against typhoid; therefore, they were considered “naïve.” However, the *S*. Typhi genome has approximately 90% homology with other *Salmonella* serovars (53), and immune cross-reactivity has been described with *S*. Paratyphi A and B as well as various non-typhoidal *Salmonella* (54). Cross-reactive responses may also be elicited by exposure to other Enterobacteriaeceae. Consequently, differences in baseline responses could be due to memory responses mounted from previous encounters with other enteric Gram negative bacilli, including those present in the normal gut microbiota (7, 55, 56). Several studies indicate that the gut microbiota plays an important role in modulating host immune responses to pathogens or to vaccination (55–57). For example, we have recently reported in healthy adults that oral administration of the Ty21a live-attenuated *S*. Typhi vaccine caused no disruption in the composition, diversity, or stability of the fecal microbiota. However, we observed that distinct multiphasic CMI responses were associated with greater community richness and diversity compared to individuals with only late CMI responses to Ty21a (56). To assess the role of the gut microbial community, we have initiated studies to identify the interplay between the host immune response, the microbiota and clinical outcome in participants challenged with wt *S*. Typhi.

In addition to these environmental factors, genetic determinants, like HLA molecules, can be critical in defining the variation in immune responses. For example, the presence of the *HLA*-DRB1*04:05 allele was recently shown to be strongly protective against *S*. Typhi (58).

In summary, these studies provided unique insights into the vast complexity of the human host immune response during the development of typhoid fever. Baseline responses of *S*. Typhi-responsive CD8+ T cells were identified as significant correlates of clinical outcome after infection. These studies also revealed some of the immunological mechanisms responsible for delayed time to disease onset and demonstrated that MF T cells are likely to play a key role in the host’s response to wt *S*. Typhi infection. Finally, this information reinforces the importance of conducting detailed CMI measurements to support the selection of future vaccine candidates for evaluation in clinical trials.

## Author Contributions

TD, CW, ML, and AP set up the challenge model and generated the clinical data. TD, CW, CJ, CB, and BA collected and processed the PBMC specimens. SF, MM, and MS conceived and designed the experiments. SF performed the experiments. SF, MM, LM, and MS analyzed and interpreted the data. All the authors contributed to the writing of the manuscript and approved the final version.

## Conflict of Interest Statement

The authors declare that the research was conducted in the absence of any commercial or financial relationships that could be construed as a potential conflict of interest.

## Abbreviations

CMI, cell-mediated immunity; T_N_, naïve T; T_CM_, T central memory; T_EM_, T effector memory; T_EMRA_, T effector memory CD45RA+.

## References

[1] Crump JA Mintz ED. Global trends in typhoid and paratyphoid fever. Clin Infect Dis (2010) 50:241–6. 10.1086/649541 20014951 PMC2798017

[2] Dougan G Baker S. *Salmonella enterica* serovar Typhi and the pathogenesis of typhoid fever. Annu Rev Microbiol (2014) 68:317–36. 10.1146/annurev-micro-091313-103739 25208300

[3] Dougan G John V Palmer S Mastroeni P. Immunity to salmonellosis. Immunol Rev (2011) 240:196–210. 10.1111/j.1600-065X.2010.00999.x 21349095

[4] Levine MM Tacket CO Sztein MB. Host-*Salmonella* interaction: human trials. Microbes Infect (2001) 3:1271–9. 10.1016/S1286-4579(01)01487-3 11755415

[5] Hornick RB Greisman SE Woodward TE DuPont HL Dawkins AT Snyder MJ Typhoid fever: pathogenesis and immunologic control. N Engl J Med (1970) 283:686–91. 10.1056/NEJM197010012831406 4916913

[6] Hornick RB Woodward TE Appraisal of typhoid vaccine in experimentally infected human subjects. Trans Am Clin Climatol Assoc (1967) 78:70–8. 6028241 PMC2441150

[7] Sztein MB Salerno-Goncalves R McArthur MA. Complex adaptive immunity to enteric fevers in humans: lessons learned and the path forward. Front Immunol (2014) 5:516. 10.3389/fimmu.2014.00516 25386175 PMC4209864

[8] Waddington CS Darton TC Jones C Haworth K Peters A John T An outpatient, ambulant-design, controlled human infection model using escalating doses of *Salmonella* Typhi challenge delivered in sodium bicarbonate solution. Clin Infect Dis (2014) 58:1230–40. 10.1093/cid/ciu078 24519873 PMC3982839

[9] Darton TC Blohmke CJ Pollard AJ. Typhoid epidemiology, diagnostics and the human challenge model. Curr Opin Gastroenterol (2014) 30:7–17. 10.1097/MOG.0000000000000021 24304980

[10] Darton TC Jones C Blohmke CJ Waddington CS Zhou L Peters A Using a human challenge model of infection to measure vaccine efficacy: a randomised, controlled trial comparing the typhoid vaccines M01ZH09 with placebo and Ty21a. PLoS Negl Trop Dis (2016) 10(8):e0004926. 10.1371/journal.pntd.0004926 27533046 PMC4988630

[11] Fresnay S McArthur MA Magder L Darton TC Jones C Waddington CS *Salmonella* Typhi-specific multifunctional CD8+ T cells play a dominant role in protection from typhoid fever in humans. J Transl Med (2016) 14:62. 10.1186/s12967-016-0819-7 26928826 PMC4772330

[12] Salerno-Goncalves R Pasetti MF Sztein MB Characterization of CD8(+) effector T cell responses in volunteers immunized with *Salmonella enterica* serovar Typhi strain Ty21a typhoid vaccine. J Immunol (1950) 169(2002):2196–203. 10.4049/jimmunol.169.4.2196 12165550

[13] Sztein MB Tanner MK Polotsky Y Orenstein JM Levine MM. Cytotoxic T lymphocytes after oral immunization with attenuated vaccine strains of *Salmonella typhi* in humans. J Immunol (1995) 155:3987–93. 7561107

[14] Salerno-Goncalves R Wyant TL Pasetti MF Fernandez-Vina M Tacket CO Levine MM Concomitant induction of CD4+ and CD8+ T cell responses in volunteers immunized with *Salmonella enterica* serovar Typhi strain CVD 908-htrA. J Immunol (1950) 170(2003):2734–41. 10.4049/jimmunol.170.5.2734 12594304

[15] Salerno-Goncalves R Fernandez-Vina M Lewinsohn DM Sztein MB Identification of a human HLA-E-restricted CD8+ T cell subset in volunteers immunized with *Salmonella enterica* serovar Typhi strain Ty21a typhoid vaccine. J Immunol (1950) 173(2004):5852–62. 10.4049/jimmunol.173.9.5852 15494539

[16] Salerno-Goncalves R Wahid R Sztein MB. Immunization of volunteers with *Salmonella enterica* serovar Typhi strain Ty21a elicits the oligoclonal expansion of CD8+ T cells with predominant Vbeta repertoires. Infect Immun (2005) 73:3521–30. 10.1128/IAI.73.6.3521-3530.2005 15908381 PMC1111837

[17] Wahid R Salerno-Goncalves R Tacket CO Levine MM Sztein MB. Cell-mediated immune responses in humans after immunization with one or two doses of oral live attenuated typhoid vaccine CVD 909. Vaccine (2007) 25:1416–25. 10.1016/j.vaccine.2006.10.040 17182155 PMC1840048

[18] Sztein MB. Cell-mediated immunity and antibody responses elicited by attenuated *Salmonella enterica* serovar Typhi strains used as live oral vaccines in humans. Clin Infect Dis (2007) 45(Suppl 1):S15–9. 10.1086/518140 17582562

[19] Wahid R Salerno-Goncalves R Tacket CO Levine MM Sztein MB. Generation of specific effector and memory T cells with gut- and secondary lymphoid tissue-homing potential by oral attenuated CVD 909 typhoid vaccine in humans. Mucosal Immunol (2008) 1:389–98. 10.1038/mi.2008.30 19079203 PMC3215293

[20] Salerno-Goncalves R Sztein MB. Priming of *Salmonella enterica* serovar Typhi-specific CD8(+) T cells by suicide dendritic cell cross-presentation in humans. PLoS One (2009) 4:e5879. 10.1371/journal.pone.0005879 19517022 PMC2691582

[21] Salerno-Goncalves R Wahid R Sztein MB. Ex vivo kinetics of early and long-term multifunctional human leukocyte antigen E-specific CD8+ cells in volunteers immunized with the Ty21a typhoid vaccine. Clin Vaccine Immunol (2010) 17:1305–14. 10.1128/CVI.00234-10 20660136 PMC2944457

[22] McArthur MA Sztein MB. Heterogeneity of multifunctional IL-17A producing *S*. Typhi-specific CD8+ T cells in volunteers following Ty21a typhoid immunization. PLoS One (2012) 7:e38408. 10.1371/journal.pone.0038408 22679502 PMC3367967

[23] Sztein MB Wasserman SS Tacket CO Edelman R Hone D Lindberg AA Cytokine production patterns and lymphoproliferative responses in volunteers orally immunized with attenuated vaccine strains of *Salmonella* Typhi. J Infect Dis (1994) 170:1508–17. 10.1093/infdis/170.6.1508 7995991

[24] Betts MR Brenchley JM Price DA De Rosa SC Douek DC Roederer M Sensitive and viable identification of antigen-specific CD8+ T cells by a flow cytometric assay for degranulation. J Immunol Methods (2003) 281:65–78. 10.1016/S0022-1759(03)00265-5 14580882

[25] Rozot V Vigano S Mazza-Stalder J Idrizi E Day CL Perreau M Mycobacterium tuberculosis-specific CD8+ T cells are functionally and phenotypically different between latent infection and active disease. Eur J Immunol (2013) 43:1568–77. 10.1002/eji.201243262 23456989 PMC6535091

[26] Seder RA Darrah PA Roederer M. T-cell quality in memory and protection: implications for vaccine design. Nat Rev Immunol (2008) 8:247–58. 10.1038/nri2274 18323851

[27] Lundin BS Johansson C Svennerholm AM. Oral immunization with a *Salmonella enterica* serovar Typhi vaccine induces specific circulating mucosa-homing CD4(+) and CD8(+) T cells in humans. Infect Immun (2002) 70:5622–7. 10.1128/IAI.70.10.5622-5627.2002 12228290 PMC128315

[28] Pasetti MF Simon JK Sztein MB Levine MM. Immunology of gut mucosal vaccines. Immunol Rev (2011) 239:125–48. 10.1111/j.1600-065X.2010.00970.x 21198669 PMC3298192

[29] Agace WW. T-cell recruitment to the intestinal mucosa. Trends Immunol (2008) 29:514–22. 10.1016/j.it.2008.08.003 18838302

[30] Mora JR von Andrian UH. T-cell homing specificity and plasticity: new concepts and future challenges. Trends Immunol (2006) 27:235–43. 10.1016/j.it.2006.03.007 16580261

[31] Stecher B Hardt WD. Mechanisms controlling pathogen colonization of the gut. Curr Opin Microbiol (2011) 14:82–91. 10.1016/j.mib.2010.10.003 21036098

[32] Barman M Unold D Shifley K Amir E Hung K Bos N Enteric salmonellosis disrupts the microbial ecology of the murine gastrointestinal tract. Infect Immun (2008) 76:907–15. 10.1128/IAI.01432-07 18160481 PMC2258829

[33] Johanns TM Ertelt JM Rowe JH Way SS. Regulatory T cell suppressive potency dictates the balance between bacterial proliferation and clearance during persistent *Salmonella* infection. PLoS Pathog (2010) 6:e1001043. 10.1371/journal.ppat.1001043 20714351 PMC2920851

[34] McArthur MA Fresnay S Magder LS Darton TC Jones C Waddington CS Activation of *Salmonella* Typhi-specific regulatory T cells in typhoid disease in a wild-type *S*. Typhi challenge model. PLoS Pathog (2015) 11:e1004914. 10.1371/journal.ppat.1004914 26001081 PMC4441490

[35] Day CL Kaufmann DE Kiepiela P Brown JA Moodley ES Reddy S PD-1 expression on HIV-specific T cells is associated with T-cell exhaustion and disease progression. Nature (2006) 443:350–4. 10.1038/nature05115 16921384

[36] Horne-Debets JM Faleiro R Karunarathne DS Liu XQ Lineburg KE Poh CM PD-1 dependent exhaustion of CD8+ T cells drives chronic malaria. Cell Rep (2013) 5:1204–13. 10.1016/j.celrep.2013.11.002 24316071

[37] Wherry EJ Blattman JN Murali-Krishna K van der Most R Ahmed R. Viral persistence alters CD8 T-cell immunodominance and tissue distribution and results in distinct stages of functional impairment. J Virol (2003) 77:4911–27. 10.1128/JVI.77.8.4911-4927.2003 12663797 PMC152117

[38] Kondo A Yamashita T Tamura H Zhao W Tsuji T Shimizu M Interferon-gamma and tumor necrosis factor-alpha induce an immunoinhibitory molecule, B7-H1, via nuclear factor-kappaB activation in blasts in myelodysplastic syndromes. Blood (2010) 116:1124–31. 10.1182/blood-2009-12-255125 20472834 PMC3375140

[39] Winter SE Winter MG Poon V Keestra AM Sterzenbach T Faber F *Salmonella enterica* serovar Typhi conceals the invasion-associated type three secretion system from the innate immune system by gene regulation. PLoS Pathog (2014) 10:e1004207. 10.1371/journal.ppat.1004207 24992093 PMC4081808

[40] Wangdi T Lee CY Spees AM Yu C Kingsbury DD Winter SE The Vi capsular polysaccharide enables *Salmonella enterica* serovar Typhi to evade microbe-guided neutrophil chemotaxis. PLoS Pathog (2014) 10:e1004306. 10.1371/journal.ppat.1004306 25101794 PMC4125291

[41] Swart AL Hensel M. Interactions of *Salmonella enterica* with dendritic cells. Virulence (2012) 3:660–7. 10.4161/viru.22761 23221476 PMC3545948

[42] van der Velden AW Copass MK Starnbach MN. *Salmonella* inhibit T cell proliferation by a direct, contact-dependent immunosuppressive effect. Proc Natl Acad Sci U S A (2005) 102:17769–74. 10.1073/pnas.0504382102 16306269 PMC1308886

[43] Atif SM Winter SE Winter MG McSorley SJ Baumler AJ. *Salmonella enterica* serovar Typhi impairs CD4 T cell responses by reducing antigen availability. Infect Immun (2014) 82:2247–54. 10.1128/IAI.00020-14 24643532 PMC4019166

[44] Ledgerwood JE DeZure AD Stanley DA Novik L Enama ME Berkowitz NM Chimpanzee adenovirus vector Ebola vaccine – preliminary report. N Engl J Med (2014). 10.1056/NEJMoa1410863 25426834

[45] Darrah PA Patel DT De Luca PM Lindsay RW Davey DF Flynn BJ Multifunctional TH1 cells define a correlate of vaccine-mediated protection against Leishmania major. Nat Med (2007) 13:843–50. 10.1038/nm1592 17558415

[46] Betts MR Nason MC West SM De Rosa SC Migueles SA Abraham J HIV nonprogressors preferentially maintain highly functional HIV-specific CD8+ T cells. Blood (2006) 107:4781–9. 10.1182/blood-2005-12-4818 16467198 PMC1895811

[47] Wolpe SD Cerami A. Macrophage inflammatory proteins 1 and 2: members of a novel superfamily of cytokines. FASEB J (1989) 3:2565–73. 2687068 10.1096/fasebj.3.14.2687068

[48] Wahid R Fresnay S Levine MM Sztein MB. Immunization with Ty21a live oral typhoid vaccine elicits crossreactive multifunctional CD8+ T-cell responses against *Salmonella enterica* serovar Typhi, *S*. Paratyphi A, and *S*. Paratyphi B in humans. Mucosal Immunol (2015) 8(6):1349–59. 10.1038/mi.2015.24 25872480 PMC4607552

[49] Bhuiyan S Sayeed A Khanam F Leung DT Rahman Bhuiyan T Sheikh A Cellular and cytokine responses to *Salmonella enterica* serotype Typhi proteins in patients with typhoid fever in Bangladesh. Am J Trop Med Hyg (2014) 90:1024–30. 10.4269/ajtmh.13-0261 24615129 PMC4047724

[50] Le Bourhis L Martin E Peguillet I Guihot A Froux N Core M Antimicrobial activity of mucosal-associated invariant T cells. Nat Immunol (2010) 11:701–8. 10.1038/ni.1890 20581831

[51] Salerno-Goncalves R Rezwan T Sztein MB. B cells modulate mucosal associated invariant T cell immune responses. Front Immunol (2014) 4:511. 10.3389/fimmu.2013.00511 24432025 PMC3882667

[52] Schenkel JM Masopust D. Tissue-resident memory T cells. Immunity (2014) 41:886–97. 10.1016/j.immuni.2014.12.007 25526304 PMC4276131

[53] McClelland M Sanderson KE Spieth J Clifton SW Latreille P Courtney L Complete genome sequence of *Salmonella enterica* serovar Typhimurium LT2. Nature (2001) 413:852–6. 10.1038/35101614 11677609

[54] Wahid R Simon R Zafar SJ Levine MM Sztein MB. Live oral typhoid vaccine Ty21a induces cross-reactive humoral immune responses against *Salmonella enterica* serovar Paratyphi A and *S*. Paratyphi B in humans. Clin Vaccine Immunol (2012) 19:825–34. 10.1128/CVI.00058-12 22492745 PMC3370435

[55] Belkaid Y Hand TW. Role of the microbiota in immunity and inflammation. Cell (2014) 157:121–41. 10.1016/j.cell.2014.03.011 24679531 PMC4056765

[56] Eloe-Fadrosh EA McArthur MA Seekatz AM Drabek EF Rasko DA Sztein MB Impact of oral typhoid vaccination on the human gut microbiota and correlations with *S*. Typhi-specific immunological responses. PLoS One (2013) 8:e62026. 10.1371/journal.pone.0062026 23637957 PMC3634757

[57] Ferreira RB Antunes LC Finlay BB Should the human microbiome be considered when developing vaccines? PLoS Pathog (2010) 6:e1001190 10.1371/journal.ppat.1001190 21124987 PMC2987818

[58] Dunstan SJ Hue NT Han B Li Z Tram TT Sim KS Variation at HLA-DRB1 is associated with resistance to enteric fever. Nat Genet (2014) 46(12):1333–6. 10.1038/ng.3143 25383971 PMC5099079

