# Supplementary material for: Importance of Salmonella Typhi-Responsive CD8+ T Cell Immunity in a Human Typhoid Fever Challenge Model
Source: Front Immunol. 2017 Mar 2;8:208. doi: 10.3389/fimmu.2017.00208 (PMC5332428; doi:10.3389/fimmu.2017.00208)
Supplement: Supplementary file 1 [file Data_Sheet_1.DOCX]

SUPPLEMENTAL TABLE AND FIGURES


Figure S1. Baseline hematological parameters and *S.* Typhi-specific responses at baseline. Total white cell count (WCC) and absolute numbers of lymphocytes were assessed by routine blood hematology before challenge. Absolute numbers of CD3+, CD3+CD8+, and CD3+CD4+ cells, and of CD3+CD8+ memory subsets (T_EM_, T_EMRA_ and T_CM_) were calculated using the percentages of positive cells obtained by flow cytometry analysis. Statistical analyses were performed using Mann-Whitney test. (B) Individual representation of baseline CD8+ T_EM_ immune responses following stimulation with each of the *S.* Typhi-infected cell types. PBMC isolated at baseline from each participant (TD n=13, blue; NoTD n=7, red) were stimulated for 18h with *S.* Typhi-infected AEH cells (squares), *S.* Typhi-infected B-EBV cells (circles) or *S.* Typhi-infected blasts (triangles). After co-culture, cells were immunostained with a 14-color panel of mAbs and analyzed by follow cytometry as described in Materials and Methods. Each symbol represents the net percentage of positive cells measured for CD107a, IFN-γ, TNF-α, MIP-1β, IL-17A and IL-2 in the CD8+ T_EM_, T_EMRA_ and T_CM_ subsets as indicated. Statistical analyses were performed using mixed effects models to account for multiple observations per person. *p< 0.05; **p< 0.01

Figure S2. Absolute numbers of T cells subsets in circulation after challenge. Shown are the kinetics of various T cell subsets in representative participants from the TD and NoTD groups. (A) Absolute numbers of CD3+, CD3+CD8+, and CD3+CD4+ cells following challenge were calculated using the percentages of positive cells obtained by flow cytometry analysis. (B) Absolute numbers of IFN-γ+ and CD107a expressing *S.* Typhi-specific CD8+ T_EM_ following challenge.

Figure S3. Kinetics and amplitude of *S*. Typhi-specific CD8+ T cell responses after challenge for all participants. (A) Kinetics of IFN-γ production by CD8+ T_EM_ following stimulation with *S.* Typhi-infected AEH are represented for each individual. (B) Areas under the curve were measured around the time of diagnosis for each biomarker in CD8+ T_EM_, T_EMRA_ and T_CM_ subsets. Each bar represents mean ± SEM of area under the curve obtained after stimulation with *S.* Typhi-infected AEH cells, B-EBV cells and blasts.

Figure S4. Absolute numbers of *S.* Typhi-specific CD8+ T_EM_ multifunctional cells and homing potential of the dominant *S.* Typhi-specific CD8+ T_EM_ multifunctional populations at baseline. (A) Absolute numbers of single and MF *S.* Typhi-specific CD8+ T_EM_ cells were calculated using the percentages of positive cells obtained by flow cytometry analysis. **(B)** Shown are the 9 major individual populations of MF integrin α_4_β_7_- and integrin α_4_β_7_+ *S.* Typhi-specific CD8+ T_EM_ cells at baseline in all TD participants. ***p< 0.001

Figure S5. Dominant populations and gut homing capabilities of MF *S.* Typhi-specific CD8+ T_EM_ responses in TD and NoTD participants after challenge. Flow cytometry data were analyzed using the FCOM function of Winlist to determine the proportion of all possible combinations of the 6 measured biomarkers to identify MF cells (i.e., positive for several biomarkers concomitantly). Percentages were measured at day 7 for NoTD participants and at 48 hours after typhoid diagnosis for TD participants. Each symbol represents the percentage of the different populations measured after stimulation with *S.* Typhi-infected cells (AEH cells [squares], B-EBV [circles] cells or blasts [triangles]) for each participant. (A) The percentages of single positive cells (1+) or of total MF cells (i.e., the sum of all cells concomitantly positive for 2 or more biomarkers) are represented for all CD8+ T_EM_ and for CD8+ T_EM_ integrin α_4_β_7_- and integrin α_4_β_7_+ cells. (B) MF cells were divided into 4 groups on the basis of the number of biomarkers they expressed. (e.g., cells expressing 2 biomarkers are shown as double positive (2+)) in CD8+ T_EM_ integrin α_4_β_7_- and integrin α_4_β_7_+ cells. (C) The 9 major individual populations of MF in CD8+ T_EM_ are represented separately for *S.* Typhi-specific integrin α_4_β_7_- (dark green) and integrin α_4_β_7_+ (purple) CD8+ T_EM_ in TD participants 48 hours after diagnosis.
